# Hepatoprotective Effect of Kaempferol: A Review of the Dietary Sources, Bioavailability, Mechanisms of Action, and Safety

**DOI:** 10.1155/2023/1387665

**Published:** 2023-02-27

**Authors:** Maulana Yusuf Alkandahri, Barolym Tri Pamungkas, Zulpakor Oktoba, Mareetha Zahra Shafirany, Lela Sulastri, Maya Arfania, Ebta Narasukma Anggraeny, Ade Pratiwi, Fitri Dwi Astuti, Indriyani, Siti Yuliani Dewi, Salsa Zulfa Hamidah

**Affiliations:** ^1^Faculty of Pharmacy, Universitas Buana Perjuangan Karawang, Karawang, West Java, Indonesia; ^2^Department of Pharmaceutical Biology, Faculty of Pharmacy, Universitas Mulawarman, Samarinda, East Kalimantan, Indonesia; ^3^Department of Pharmacy, Faculty of Medicine, Universitas Lampung, Bandar Lampung, Indonesia; ^4^Department of Pharmaceutical Biology, Sekolah Tinggi Farmasi Muhammadiyah Cirebon, Cirebon, West Java, Indonesia; ^5^Department of Pharmaceutics and Pharmaceutical Technology, Sekolah Tinggi Farmasi Muhammadiyah Cirebon, Cirebon, West Java, Indonesia; ^6^College of Pharmaceutical Sciences, Yayasan Pharmasi Semarang, Semarang, Central Java, Indonesia; ^7^Student of Pharmacy, Faculty of Pharmacy, Universitas Buana Perjuangan Karawang, Karawang, West Java, Indonesia

## Abstract

The liver is the body's most critical organ that performs vital functions. Hepatic disorders can affect the physiological and biochemical functions of the body. Hepatic disorder is a condition that describes the damage to cells, tissues, structures, and functions of the liver, which can cause fibrosis and ultimately result in cirrhosis. These diseases include hepatitis, ALD, NAFLD, liver fibrosis, liver cirrhosis, hepatic failure, and HCC. Hepatic diseases are caused by cell membrane rupture, immune response, altered drug metabolism, accumulation of reactive oxygen species, lipid peroxidation, and cell death. Despite the breakthrough in modern medicine, there is no drug that is effective in stimulating the liver function, offering complete protection, and aiding liver cell regeneration. Furthermore, some drugs can create adverse side effects, and natural medicines are carefully selected as new therapeutic strategies for managing liver disease. Kaempferol is a polyphenol contained in many vegetables, fruits, and herbal remedies. We use it to manage various diseases such as diabetes, cardiovascular disorders, and cancers. Kaempferol is a potent antioxidant and has anti-inflammatory effects, which therefore possesses hepatoprotective properties. The previous research has studied the hepatoprotective effect of kaempferol in various hepatotoxicity protocols, including acetaminophen (APAP)-induced hepatotoxicity, ALD, NAFLD, CCl_4_, HCC, and lipopolysaccharide (LPS)-induced acute liver injury. Therefore, this report aims to provide a recent brief overview of the literature concerning the hepatoprotective effect of kaempferol and its possible molecular mechanism of action. It also provides the most recent literature on kaempferol's chemical structure, natural source, bioavailability, and safety.

## 1. Introduction

The liver is a very important organ because of the many physiological processes it controls. A number of critical processes, including secretion, metabolism, and storage, are linked to these actions. In addition to synthesis, this organ also has the ability to detoxify both endogenous (metabolic waste) and external (toxic chemicals) toxins [1]. In addition to its role in digestion, the liver is involved in the biochemical processes of development, nutrition, energy production, and reproduction. It contributes to carbohydrate and fat metabolism, bile secretion, and vitamin storage [2]. The liver disease is still one of the significant threats to public health and a global problem because of these functions [3]. When the cells, tissues, structures, and activities of the liver are damaged, it is called liver disease. This damage can be brought on by microorganisms (bacteria, viruses, and parasites) and autoimmune conditions (immune hepatitis and primary biliary cirrhosis). Toxic substances such carbon tetrachloride (CCl_4_), thioacetamide, dimethylnitrosamine (DMN), and D-galactosamine/lipopolysaccharide (D-GalN/LPS), as well as medications such as paracetamol and antituberculosis drugs in large dosages [4–6], can also cause this harm. Despite the advances in modern medicine, no drug is fully effective in stimulating the liver function, offering complete protection, and aiding hepatocyte regeneration [7, 8]. Therefore, it is essential to identify alternative drugs to treat liver disease, indicating that these agents are more effective and less toxic. Natural products provide a treasury for discovering new compounds in treating various diseases such as cancer, inflammation, and liver disease. More than half of pharmaceutical products are from natural compounds and their derivatives [9]. Around 80% of patients with liver disease use herbal treatments because they are easy to find, have low toxicity, have pharmacological activity and chemical variety, and have common side effects compared to synthetic drugs [10]. Kaempferol is one of the most common aglycone flavonoids in the form of glycosides. The four hydroxy groups of this yellow molecule are located at the 3, 5, 7, and 4′ positions, making it a tetrahydroxyflavone [11]. It is found in a wide variety of plant foods and plant-based supplements, including kale, beans, tea, spinach, and broccoli [12, 13]. Cardioprotective, neuroprotective, anti-inflammatory, antidiabetic, antioxidant, antitumor, and anticancer effects have been seen with kaempferol and its glycosylated derivatives [14, 15]. In light of these data, this article is to compile and discuss the effects of kaempferol on the prevention and treatment of liver disease, with a focus on its molecular mechanisms of action.

## 2. Materials and Methods

This is a nonsystematic review article using electronically based data. This is a nonsystematic review that was conducted using electronic databases such as “Scopus,” “PubMed,” “MEDLINE,” “Science Direct,” “Cochrane library,” and “Web of Science” to search for cellular, animal, or human studies with the keywords “kaempferol,” “hepatoprotective, hepatotoxicity, or liver,” “hepatocellular carcinoma,” “liver fibrosis process,” “liver cirrhosis,” and “cellular and molecular mechanisms of liver fibrosis”. This study evaluates all articles written in English that include *in vitro* experiments, animal models, and human-related data. Articles with incomplete text, abstracts, and those published before the year 2000 are not included in this study since they do not meet the inclusion requirement.

## 3. Results and Discussion

### 3.1. Chemistry of Kaempferol

**Figure 1:**
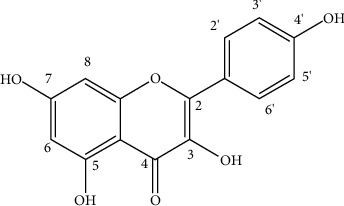
Structure of kaempferol.

Kaempferol is a polyphenol present in several fruits and vegetables (Figure 1) and drinks derived from plants. It is used to treat a variety of disorders; however, there are no review publications that describe its natural sources and hepatoprotective biological actions.

### 3.2. Natural Sources of Kaempferol

Distributed kaempferol is widespread in the food, beverage, and plant kingdoms, and the derivatives are synthesized in plants by various types of enzymes.  Table 1 summarizes the different food sources.

### 3.3. Bioavailability of Kaempferol

**Figure 2:**
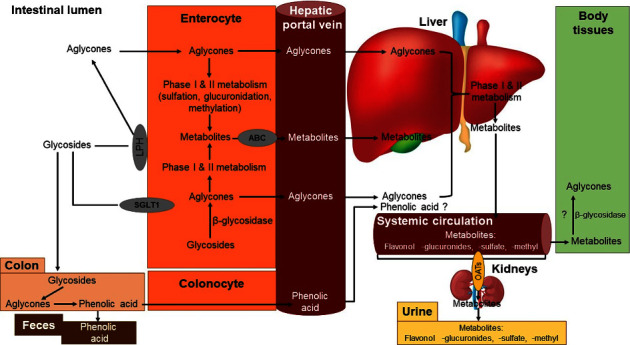
General overview of dietary kaempferol bioavailability.

Pharmacokinetic investigations have shown that high-polarity glycosides such as kaempferol have poor absorption in comparison to medium-polarity glycosides [16]. Depending on how well lipophilic aglycones diffuse passively from the intestinal lumen into enterocytes, they are either absorbed directly into the hepatic portal vein or treated before absorption [18]. Compounds generated in the first two phases of aglycones metabolism in enterocytes (O-demethylation and glucuronidation) are transferred to the liver via the ATP-binding cassette (ABC) [18, 19]. Before being taken into the circulation, lipophobic glycosides must be converted to the aglycone form in the intestinal lumen or enterocytes [20]. Glucose is thought to be converted into aglycone near the brush boundary of the stomach by the LPH enzyme [17]. Also, sodium-dependent glucose transporter 1 (SGLT 1) can metabolize glycosides brought into enterocytes via cytosolic-glucosidase [20, 21]. The aglycones produced by phase I and phase II metabolism are absorbed by ABC transporters in the hepatic portal vein [18, 19]. Serum albumin carries the metabolites of ingested aglycones to the liver [16]. Phase I and II metabolism in the liver converts leftover aglycones into methyl, sulfur, and glucuronide metabolites that are subsequently carried through the bloodstream to all of the body's organs [17]. Kaempferol has been shown to be present in plasma at nanomolar concentrations following oral ingestion in several studies. The kaempferol found in endive is also administered to eight healthy volunteers (246 mg kaempferol per kilogram of endive). The high plasma concentrations of 100 nM of kaempferol-3-glucuronide (79%) and 3-glucoside (14%) and 3-(6-malonyl)-glucoside (7%) were observed 5.8 hours after oral administration of endive containing 8.65 mg of kaempferol [22]. Hydrolysis of conjugated substances can be accomplished by an enzyme that is poorly understood in the human body despite the fact that it has been studied *in vitro* [23]. Kaempferol metabolites are excreted in urine and bile. One point nine percent (1.9%) and two point five percent (2.5%) of the total dose are eliminated through urine and bile, respectively [22]. An organic anion-delivering polypeptide (OAT) is responsible for transporting a chemical from blood to the kidneys [24, 25]. There are three types of transporter OATs: those that specialize in transporting the metabolites of the liver (glucuronide, methyl, and sulfate). The metabolites of bile are excreted in the feces or the small intestine [26]. Some glycosides are poorly absorbed in the small intestine and make it all the way to the large intestine, where they are used by the colonic microbiota [27]. Three of the more notable byproducts are 3,4-dihydroxyphenylacetic acid, 3,4-dihydroxybenzoic acid, and 3-hydroxyphenylacetic acid [16].  Figure 2 shows that these chemicals are either passed out of the body in the feces or taken into the bloodstream for further processing [26, 28]. Phenolic acid metabolites' bioactivity and their final resting place are still unknown [17].

### 3.4. Liver Disease: Pathophysiology and Epidemiology

Many factors contribute to liver disease, including fibrosis, but the final consequence is always the same: a diminished liver's ability to function. Some stimuli, such as alcohol and viral hepatitis, have the potential to cause damage more quickly or more slowly than other stimuli. The parenchyma is replaced by nodules that renew as the hepatocytes die off and an extracellular fibrotic scar forms [29, 30]. A dynamic process, fibrosis can be reversed in its early stages since it is reversible. Medicine, on the other hand, can neither stop nor reverse this process, making it impossible to forecast [31]. Fibrosis is caused by the activation of hepatic stellate cells, which is the major mechanism. There are two steps to this activation. The body initiates the first phase, which is called the initiation phase or the proinflammatory phase. In this condition, apoptosis (cell death) is induced, oxidative stress is present, and Kupffer cells, hepatocytes, platelets, and the endothelium all participate. When a combination of cell proliferation, fibrogenesis, and an inflammatory response occurs, the perpetuation phase starts [32]. A major role for metalloproteinases in the breakdown of the extracellular matrix is played by stellate cells. A lack of equilibrium between the body's pace of creation and its breakdown causes damage to be replaced by fibrosis [33]. The overly damaged matrix can be reabsorbed and reverse hepatic alterations, which is why novel therapies are focused on this process [34].

Anyone, regardless of gender or ethnicity, can be affected by liver disease, which is a long-term ailment. It is also dispersed unevenly across the wide range of countries where it may be found [35]. One million fatalities per year are caused by cirrhosis complications, while the other million are caused by viruses, hepatitis, and liver cancer. 3.5% of all global deaths are caused by cirrhosis and liver cancer. Liver cancer is the 16^th^ most common cause of death, whereas cirrhosis is currently the 11^th^ most common [6]. The drinking habits of more than 75 million individuals throughout the world have been linked to alcoholism, putting them at risk of developing liver disease [36]. According to the World Health Organization, there are approximately 400 million diabetics worldwide. Having either of these problems may result in NAFLD or HCC [37, 38]. Despite this, drug-induced liver damage remains the predominant cause of acute viral hepatitis [6]. Although the frequency is underestimated by as much as 5–7% annually [39], it is present in asymptomatic, undiagnosed individuals who eventually advance to decompensated phases.

### 3.5. Hepatoprotective Potential of Kaempferol

Its antidepressant, anxiety-reducing, anti-inflammatory, and antitumor properties are just a few of the many that make kaempferol so unique [15, 40]. A number of previous investigations have shown that this chemical has hepatoprotective properties [41]. Pretreatment with kaempferol in CCl_4_-induced rats corrected the hepatic enzyme activity, and it reduced liver damage in rats treated with acetaminophen via boosting the SIRT1 activity [42]. Antioxidant, anti-inflammatory, and antiapoptotic actions of SIRT1 are suppressed by inhibiting acetylation of all SIRT1 targets such aPARP1, p53, NF-*κ*B, and FOXO-1 [43]. Hepatoprotective effects of kaempferol on alcohol-induced liver damage in rats can be achieved via lowering CYP2E1 expression and increasing the antioxidant defense system's protective role [44]. Furthermore, it induces selective cytotoxicity in HCC hepatocytes [45], suppresses lipopolysaccharide-induced acute liver injury [46], and can act as an antifibrotic agent for liver fibrosis by selectively binding to ALK5, which further downregulates the TGF-*β*/Smads pathway [47].  Table 2 summarizes the various studies reporting the role of the compound in managing severe liver injury.

#### 3.5.1. Kaempferol Suppresses Liver Damage by the Upregulation and Activation of SIRT1

Acetaminophen (APAP)-induced hepatotoxicity causes 500 deaths and >80,000 the emergency room visits annually [43]. Inflammation, mitochondrial damage, ROS, necrosis, and apoptosis are the most well-described pathways by which APAP causes liver injury [57]. NAPQI is a hepatotoxic metabolite of APAP in a well-functioning liver [58]. NAPQI is transformed into a less harmful form by attaching to glutathione (GSH) in cells [59]. Yet, high hepatic APAP levels lower intracellular GSH levels, leading to oxidative stress, excessive ROS production [60], and hepatic apoptosis [57]. Multiple studies over the past decade have established the crucial protective role of SIRT1 in the liver after APAP intoxication. Cell survival, antioxidant levels, and apoptosis are all influenced by SIRT1, which has several biological functions in most cells, including liver cells [61]. NAD + -dependent deacetylase primarily mediates this effect by deacetylating several transcription factors (including NF-*κ*B and STAT3) involved in inflammation, antioxidant potential, and cell survival, fork-head transcription factors (FOXOs and p53), and PGC-1 involved in mitochondrial biogenesis [62]. By deacetylating a number of transcription factors, SIRT1 increases cell survival and proliferation, reduces cellular inflammation and oxidative stress, and boosts mitochondrial biogenesis and ATP production [63]. After being exposed to APAP, the amount of APAP in the livers of both humans and rodents drops dramatically. Conversely, SIRT1 activation by pharmaceutical means is very protective [64]. It suggests that a medicine that protects the liver from toxicity can be used as a treatment method to counteract the effects of APAP. By increasing antioxidants and decreasing inflammation and apoptosis in multiple organs, including the brain, liver, kidney, and heart, food crops containing kaempferol can provide comprehensive protection against oxidative organ damage and numerous deadly illnesses [65].

**Figure 3:**
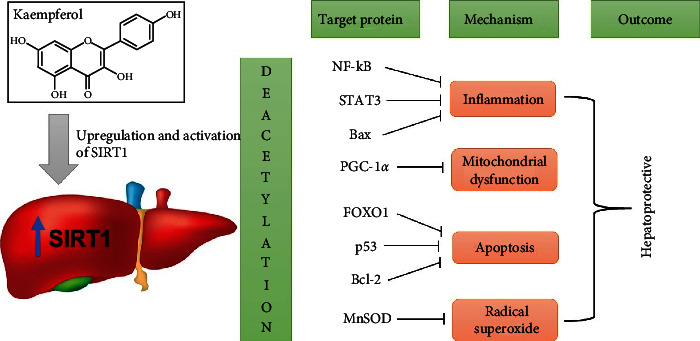
Upregulation and activation of SIRT1 in the liver by kaempferol. The SIRT1 deacetylase activity and expression increases, which leads to inhibition of several transcription factors, such as inflammation, mitochondrial dysfunction, apoptosis, and radical superoxide, in the liver tissue. Inhibition (⟞).

BinMowyna and AlFaris stated that the hepatoprotective effect of the compound involves the upregulation and activation of SIRT1 and deacetylation of FOXO1. It suppresses the expression of certain apoptotic genes while increasing the expression of antioxidant and antiapoptotic genes (MnSOD and Bcl-2), inhibits p53 acetylation, nuclear translocation, and subsequent Bax synthesis, as well as deacetylates and inactivates NF-*κ*B p65, which generally stimulates inflammation by upregulation of inflammatory cytokines and induces apoptosis through upregulation of Bax and inhibition of Bcl-2 [43] (Figure 3). This effect shows that kaempferol plays a role in regulating apoptotic mediators [66].

#### 3.5.2. Kaempferol Suppresses the Hepatic Activity of CYP2E1

High alcohol intake is related with oxidative stress, which causes hepatotoxicity by increasing reactive oxygen species, such as ROS, and decreasing antioxidant defenses [67]. Furthermore, oxidative stress and lipid peroxidation are crucial in alcohol-induced liver toxicity. Ethanol can be metabolized to ROS by alcohol dehydrogenase (ADH) and CYP2E1 [68]. ROS are released extensively, triggering toxic effects directly or indirectly through lipid peroxides [69]. A recent study reported that ethanol-induced liver injury and lipid peroxidation correlate with the CYP2E1 activity [70]. Several markers are due to the induction of oxidative stress, including increased liver enzyme levels (AST and ALT) and intracellular calcium levels. Increased AST and ALT levels can damage liver cells [71], and as pyridoxal phosphate (PLP) dependent enzymes, they can initiate the conversion of aspartate and ketoglutarate to glutamate and oxaloacetate. Increased levels of these enzymes in the liver may occur in response to cellular damage, with decreased integrity at the functional level of the membranes [72]. The report also stated that oxidative stress induces Nrf2 in human hepatocyte cells by permitting the dissociation from Keap1 and translocation into the nucleus, which attaches to antioxidant response elements and leads to the expression of target genes [71].

**Figure 4:**
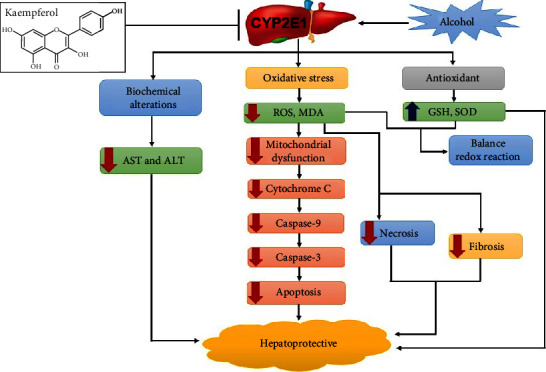
Kaempferol could protect the alcohol-induced hepatotoxicity by inhibiting the CYP2E1 expression and activity. The red arrow indicates the downregulated genes, metabolites, or enzymes. The blue arrow indicates the upregulated genes, metabolites, or enzymes. Inhibition (⟞).

Inhibiting alcohol-mediated activation of CYP2E1 can reduce liver injury caused by oxidative stress and lipid peroxidation. Several CYP2E1 inhibitors cannot protect against liver injury [73]. The report stated that kaempferol could inhibit CYP2E1 at both expression and activity levels to cause a decrease in ROS (H_2_O_2_ and MDA) [74]. A remarkable decrease in serum AST and ALT levels follows this inhibitory effect [75]. The compound can also induce reactive antioxidant enzymes (GSH and SOD) for the clearance of lipid products (MDA) and ROS (H_2_O_2_) [44]. Meanwhile, the protective effect on the liver structure occurs because kaempferol can inhibit hepatocyte apoptosis by reducing the apoptosis-related proteins expressions, including cytochrome c, Bax, Bcl-2, caspase-3, caspase-8, and caspase-9 (Figure 4) [76].

#### 3.5.3. Selective Cytotoxicity of Kaempferol on Cancerous Hepatocytes

Hepatocellular carcinoma (HCC) is the most prevalent kind of primary liver cancer in adults and the second greatest cause of mortality [77]. Surgical resection and local ablative therapy are adopted when liver transplantation is inaccessible, and the recurrence is the leading reason for death after treatment for this cancer [78]. Chemotherapy is an additional treatment for hepatocellular carcinoma; however, HCC has demonstrated strong resistance to many therapies [79]. Treatment of cancer remains a formidable obstacle in the medical community. Consequently, the discovery of effective natural medicines with anti-HCC properties is unquestionably crucial [80]. The use of flavonoid chemicals in the treatment of many forms of cancer, such as liver cancer, is given considerable study. Multiple investigations have demonstrated that these chemicals are present in medicinal plants and can be consumed. According to an epidemiological investigation, these natural chemicals play a significant effect in preventing carcinogenesis [81]. It has been demonstrated that kaempferol, a common flavonoid found in vegetables, fruits, and medicinal plants, has several biological functions, including anticancer properties [45]. The anticancer impact has been examined *in vitro* and has been shown to cause apoptosis in tumor cells [82, 83]. Another study reported that kaempferol is much less toxic to normal cells than standard chemotherapy drugs [84].

According to Seydi et al., kaempferol can produce specific cytotoxicity in HCC hepatocytes. The cytotoxic impact on HCC hepatocytes may be dependent on the location and replacement of hydroxyl groups in the molecule's core [45]. Under certain conditions, these molecules can behave as pro-oxidants [85]. The pro-oxidant situation is determined by the overall amount of OH groups in the molecule, the participation of free transition metal ions in the oxidation process, and the flavonoid content [86]. Kaempferol produces selectively enhanced ROS production and oxidative stress in HCC-affiliated hepatocytes. Therefore, the location and concentration of the hydroxyl group can affect ROS production [45]. Several studies indicate that ROS are involved in cancer cell apoptosis signaling. Specifically, drug-induced apoptosis of cancer cells is caused by enhanced ROS production in the targeted tumor [87]. However, there is evidence that ROS might exacerbate the drop in MMP if the medication induces apoptosis in cancer cells [88]. The reduction in MMP following disruption of the mitochondrial outer membrane by the stimulation might result in the release of cytochrome c from mitochondria, triggering apoptosis [89].

**Figure 5:**
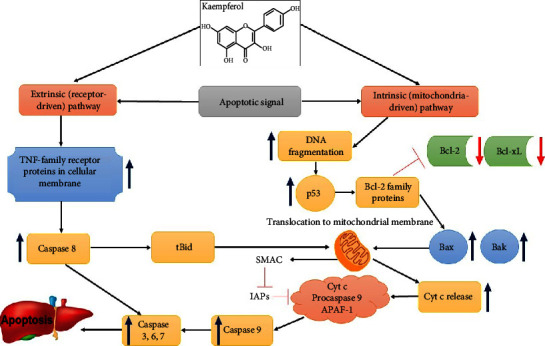
Kaempferol targets in extrinsic and intrinsic apoptosis pathways. Blue arrows show the effect of kaempferol (activation) and red arrows show suppression, inhibition (⟞).

According to several research, some medications targeting mitochondria have the ability to selectively destroy cancer cells (oxidation therapy) in preclinical and clinical trials without damaging normal cells. Owing to oxidative stress and consequent death, cancer cells are more sensitive to irreversible damage [90]. Moreover, apoptosis is vital for controlling the number of cells under diverse developmental, physiological, and pathological situations. According to several studies, the majority of malignant tumors are resistant to apoptosis [91], which is produced by a range of signal transduction pathways and proapoptotic proteins, including caspases and Bcl-2 family members [92]. There are two major signaling cascades involved in apoptosis: one is extrinsic and involves the TNF superfamily and its primary signaling protein, caspase 8, and the other is intrinsic and involves the mitochondrial route, where proteins from the Bcl-2 family initiate the activation of caspases 9, 3, and 7 [93]. A consequence of this is the upregulation of antiapoptotic proteins of the Bcl-2 family and the downregulation of proapoptotic proteins and caspases in cancer cells, as well as the overexpression of oncogenic genes that promotes cellular proliferation and inhibits p53 [93, 94]. Kaempferol is a pro-oxidant that activates B cells and blocks the growth of cancer cells by blocking the activity of EGFR/MAPK receptors, PI3Ks, and protein kinase B (Akt) [92, 95, 96]. It can specifically attack the signaling pathways that lead to cell death known as apoptotic signaling cascades [92, 97]. In addition, it prevents proliferation, differentiation, and NF-*κ*B activation by blocking Bcl-2, Bcl-xL, c-IAP1, survivin, and apoptosis [98–100]. It upregulates p53, activates caspases 3, 7, and 9, activates Bax and Bid, and downregulates Bcl-2 and Bcl-xL protein expression, as shown in Figure 5 [45, 101].

#### 3.5.4. Kaempferol Suppresses Acute Liver Injury Induced by LPS

**Figure 6:**
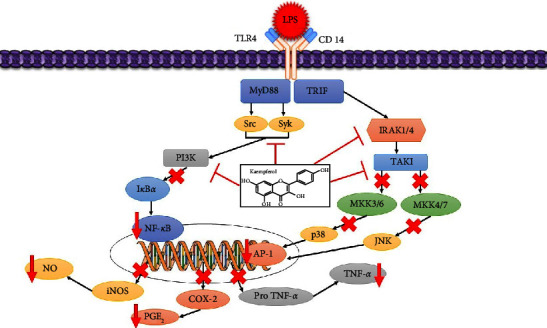
A proposed schematic diagram illustrating the mechanism of protection of kaempferol against LPS-induced acute liver injury. Inhibition (⟞), red arrows (suppression).

The liver plays a vital role in pathogen clearance and immunological reactions [102] and is susceptible to toxic chemical compounds, causing acute liver injury (ALI) [46]. Persistent liver damage can result in fibrosis and dysfunction [103], partly caused by lipopolysaccharide (LPS). LPS can induce liver injury [104] and activate receptors such as TLR4 in hepatocytes and Kupffer cells [105]. It can also promote phosphorylation of NF-*κ*B and production of proinflammatory cytokines, such as IL-6, TNF-*α*, and IL-1, to worsen inflammatory liver damage [106]. In addition, LPS-induced hepatocellular damage induces oxidative and nitrosative stress, which leads to a rise in oxidants such as oxygen ROS and RNS, a decrease in endogenous antioxidants such as SOD and GSH, and an increase in MDA [107–109]. LPS can cause Kupffer cells in the liver to release inflammatory cytokines via TLR4 [110], which is crucial for inflammation-induced liver damage [104]. Many cells initiate distinct intracellular signaling cascades through two major adapter molecules containing the generated Toll/IL-1, TRIF, and MyD88 receptor domains to activate inflammatory regulatory transcription factors, including NF-𝜅B and AP-1, to express inflammation-mediated genes encoding iNOS, COX-2, cytokines, and chemokines [111, 112]. The report found that kaempferol decreases the expression level of mRNA and the TLR4 protein significantly to inhibit the phosphorylation of NF-*κ*B p65 in liver tissue [46, 106]. It can also inhibit the production and expression of TNF-*α*, IL-1*β*, and IL-6 mRNA and COX-2 as an essential inflammatory mediator in the pathological process of inflammation [46, 113]. This compound can also significantly inhibit NO and PGE2 production and downregulate iNOS mRNA expression levels in the ALI liver tissue [46]. These results show that kaempferol can reduce liver inflammation by blocking TLR4 and NF-*κ*B activation and inhibiting the production of proinflammatory cytokines. It has protective activity against hepatic nitrosative stress and can fix liver dysfunction (Figure 6) [46, 106]. However, several studies stated that kaempferol could directly suppress the kinase activity of Src, Syk, PI3K, IRAK1, IRAK4, and TAK1 [114, 115]. This suppressive activity is associated with the suppression of subsequent downstream pathways consisting of I*κ*B*α* or MKK3/4, JNK, and p38 in NF-*κ*B modulation and AP-1 activation [111, 116, 117].

#### 3.5.5. Kaempferol Downregulates the Phosphorylation of Smad2 and Smad3

Liver fibrosis is the outcome of chronic or repetitive liver damage produced by hepatotoxic substances such as alcohol and chronic liver illnesses such as alcoholic hepatitis, hepatic steatosis, viral hepatitis infections, and autoimmune disorders [118]. Cirrhosis, which is frequently linked with liver failure, portal hypertension, and HCC [119], is the end outcome of chronic fibrosis. Generally, dysfunctional hepatic stellate cells (HSCs) play a significant role in liver fibrogenesis [120]. Under normal physiological settings, HSCs operate as vitamin A stores in their inactive form. Upon activation, however, these cells develop into myofibroblastlike cells, express *α*-SMA, and produce copious quantities of collagen [121]. When additional collagen accumulates, normal liver parenchyma is replaced by scar tissue, resulting in hepatic fibrosis [47]. TGF-*β* is a crucial regulatory cytokine throughout the liver fibrosis progression. It is known to have critical impacts on liver fibrosis, including the activation and proliferation of HSCs and the creation of the extracellular matrix (ECM) [122, 123]. TGF-*β* can bind to its cognate receptor (TGF-*β* type II) and phosphorylate Smad2 and Smad3 in order to activate HSCs and initiate transcription of profibrosis genes [124].

**Figure 7:**
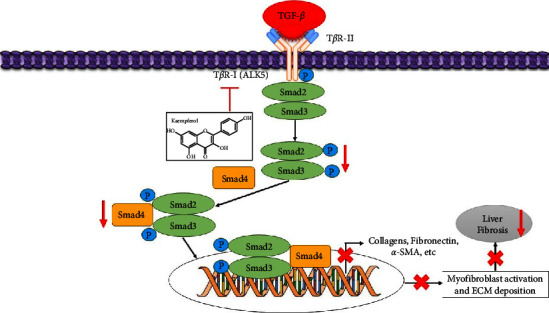
Schematic diagram depicting possible mechanisms kaempferol targets in the T*β*R-1 (ALK5) signaling pathway and downregulates the phosphorylation of Smad2 and Smad3. Inhibition (⟞) and red arrows (suppression).

Kaempferol can inhibit the expression of type I collagen in HSCs and diminish the collagen density in the liver tissue. It reduces Smad2 and Smad3 phosphorylation by the serine/threonine kinase domain, attenuates *α*-SMA production, and inhibits TGF-*β* stimulated HSCs (Figure 7). Additionally, it can bind specifically to ALK5 and further inhibit the TGF-*β*/Smads pathway [47]. It may also function as an antifibrotic agent against liver fibrosis and other fibrotic illnesses.

#### 3.5.6. Effect of Kaempferol on the NF-*κ*B Signaling Pathway in NAFLD

An estimated 25% of the global population suffers from nonalcoholic fatty liver disease (NAFLD), also known as metabolic-associated fatty liver disease (MAFLD) [125]. From simple steatosis (SS) through nonalcoholic steatohepatitis (NASH), fibrosis, cirrhosis, and HCC, NAFLD encompasses a vast spectrum of diseases. NAFLD, according to the accepted nomenclature, includes both nonalcoholic fatty liver (NAFL) and nonalcoholic steatohepatitis (NASH) [126]. Hepatic steatosis affecting more than 5% of the parenchymal tissue without signs of hepatocyte damage is a diagnostic factor of NAFL. In contrast, nonalcoholic steatohepatitis (NASH) is a necroinflammatory disease in which steatosis leads to injury of liver cells [127]. However, NAFLD is a major risk factor for many other illnesses that lead to physical health issues, such as atherosclerosis and type 2 diabetes [128]. Insulin resistance, hormone release from the adipose tissue, dietary variables, gut microbiota, genetics, and epigenetics are all linked to the onset and progression of NAFLD [129]. NAFLD therapy should aim to lower the severity of hepatic steatosis, insulin resistance, inflammation, and oxidative stress. NAFLD therapy is traditionally focused on modifying the patient's lifestyle through measures including calorie restriction, weight loss, and exercise programs. But many patients struggle with trying to make lifestyle changes. Also, no medicines for NAFLD have been authorized by the FDA [130]. Clinical trials have shown that certain medicines, including statins and pioglitazone, are effective against NAFLD; nevertheless, there are a number of drawbacks associated with using these treatments to treat NAFLD, including their single-target qualities and significant side effects [131].

**Figure 8:**
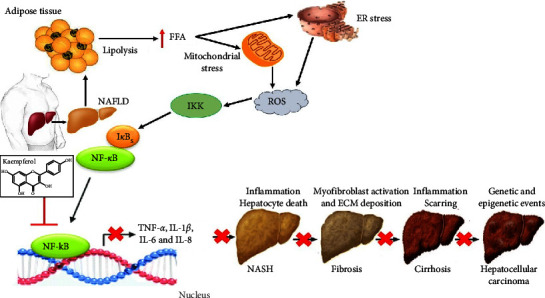
A proposed schematic diagram illustrating the mechanism of the effect of kaempferol on the NF-*κ*B signaling pathway in NAFLD. Inhibition (⟞).

Due to its multitarget qualities and potential for treating and preventing NAFLD, kaempferol has recently garnered a lot of interest. Kaempferol has been found in recent research to suppress the NF-*κ*B pathway in HepG2 cells, therefore halting the development of simple steatosis into nonalcoholic steatohepatitis [55]. In cells that regulate apoptosis and stress responses, as well as those engaged in inflammation and immunity, NF-*κ*B plays a crucial role as a nuclear transcription factor [132]. The cytoplasmic release of NF-*κ*B allows it to reach the nucleus and initiate the production of genes such as TNF-*α* and IL-6 [133]. It has been shown that TNF-*α* and IL-6, two inflammatory mediators produced in the liver of NASH patients, can engage in and mediate the inflammatory response in the pathophysiological process of NAFLD (Figure 8) [134, 135]. According to the study, TNF-*α* promotes hepatic cell degeneration and necrosis by triggering an inflammatory response in the organ. Meanwhile, IL-6 has been linked to liver cell necrosis and may play a role in NASH pathogenesis by inducing hormone resistance [136]. Because of this, blocking the NF-*κ*B signal transduction pathway may be an effective treatment strategy for halting the development of NAFLD into NASH [55, 137].

### 3.6. Safety of Kaempferol

Mutagenic and genotoxic effects of kaempferol have been observed [16]. This substance has an antioxidant action, operating as a pro-oxidant and playing a crucial part in the genotoxic impact [138]. By giving up a hydrogen atom, it neutralizes free radicals and produces phenoxyl radicals. When the second radical reacts with a phenoxyl radical, an antioxidant is produced. In contrast, the phenoxyl radical works as a pro-oxidant when it combines with oxygen species, reducing copper and iron ions that are crucial to lipid peroxidation and the generation of hydroxyl radicals [139, 140]. Researchers have shown that the CYP1A1 enzyme is responsible for the carcinogenic impact of kaempferol by converting it to the genotoxic quercetin [16]. Meanwhile, the substance is not recommended for individuals who are lacking in folic acid or iron because of its poor cellular absorption and bioavailability [141, 142]. Etoposide treatment is likewise not recommended for cancer patients using kaempferol because of the potential for interference with bioavailability [143]. Consuming kaempferol-rich foods yielding 8.04 mg/day is related with favorable benefits and no reported concern, whereas the typical dietary intake is 5.4 mg/day [144, 145].

## 4. Conclusion

Numerous research suggested that kaempferol might be a useful medication for treating liver disease. The current review evaluated previous research on the preventive benefits and mechanisms of kaempferol in various liver disorders. The liver's ability to defend the body is likely tied to its ability to regulate metabolic homeostasis, dampen inflammatory reactions, and turn on apoptotic signals. Numerous investigations have pinned down the molecular mechanism by which kaempferol works to treat liver disease, but its principal target has not been identified. There are not many clinical research on kaempferol and liver disease, despite the compound's promising results in cellular or animal models of the condition. There must immediately be a thorough investigation of the effectiveness, safety, and toxicity of this treatment in human beings. However, kaempferol's limited bioavailability and poor water solubility prevent it from being widely used in clinical applications. Changes and improved formulations are required to address the issues and boost effectiveness. Therefore, kaempferol and its derivatives will become promising drugs for treating liver disease.

## Figures and Tables

**Table 1:** Dietary sources of kaempferol.

	Food/plant/beverages	Doses (mg/kg)	References
Kaempferol	Brinjal	80	[16]
	Carrot	140	
	Papaya shoots	453	
	Green chili	39	
	Black tea	118	
	Pumpkin	371	
	White radish	38	
	Beans	14	
	Broccoli	72	
	Cauliflower	270	
	Onion leaves	832	
	Gooseberry red	19	
	Strawberry	5–8	
	Gooseberry yellow	16	
	Apples	1.4	[17]
	Asparagus	14	
	Chinese cabbage	225	
	Kale	470	
	Lettuce	8.4	
	Leeks	26.7	
	Spinach	550	
	Chives	125	
	Dill	400	
	Fennel leaves	65	
	Blueberry	31.7	
	Cherry	51.4	
	Cranberry	2.1	

**Table 2:** The role of kaempferol in alleviating liver diseases.

Disease types	In vitro/in vivo model	Mechanism of action	Concentrations/doses	References
Liver injury	Bosentan-induced rat liver injury model and HEK-293 cells	Inhibition of OATP1B1 transporter and maintaining a level of AST and ALT	25 mg/kg and 1–150 *μ*M	[48]
Liver injury	Male ddY mice	Decreased TBARS and TNF-*α* levels in CCl_4_-treated mice	4.9 mg/kg	[42]
Liver injury	Male swiss albino rats	Inhibition of lipid peroxidation caused by CCl_4_ reactive free radicals	25 mg/kg	[49]
Liver injury	Mice and HepG2 cells	Reduced AA + Fe-induced ROS production, reversed glutathione depletion, and cell death	250 and 500 mg/kg and 100, 200, and 400 *μ*M	[50]
Alcoholic liver injury	ALI mice model	Increased antioxidant defense activity and decreased oxidative stress and lipid peroxidation	10 and 20 mg/kg	[44]
Liver fibrosis	L02, LX2, and rats	Decreased protein levels of cleaved caspase-3 and increased p-ERK1/2, PI3K, and Bcl-xL protein expression in TNF-*α*-stimulated L02 cells. The suppressed proliferation of LX2 cells and upregulation of Bax and cleaved caspase-8	20 *μ*M	[51]
Liver fibrosis	HSCs/CCl_4_-induced mouse model	Downregulation of hyaluronic acid, ALT, AST, and Smad2/3. Inhibits collagen synthesis and activation of HSCs cells. Suppression of activin receptorlike kinase 5	2–10 *μ*mol/L	[47]
Liver cancer	HepG2 cells	Increases the PIG3 level at mRNA and the protein level, increases ROS production and cytochrome c release, decreases mitochondrial membrane potential, upregulation of Bax/Bcl-2, activation of caspases-9and-3, and maintains the pro-oxidant activity	10, 20, 40, and 80 *μ*M	[52]
Liver cancer	HepG2 cells	Apoptosis, reduced expression of miR-21, and upregulation of PTEN expression and the PI3K/AKT/mTOR signaling pathways inactivation	25, 50, 75, and 100 *μ*M	[53]
Hepatotoxicity	Male C57BL/6 mice	Decreased level of ALT and AST. Induce hepatocellular damage, increases expression of antioxidant enzymes, and apoptosis. Reduces the NLRP3 expression and proinflammatory factors. Inhibition of the HMGB1/TLR4/NF-*κ*B signaling pathway	30 and 60 mg/kg	[54]
Nonalcoholic fatty liver disease (NAFLD)	HepG2 cells	Decreases hepatic lipid accumulation, inhibition of the NF-*κ*B signal transduction pathway and promotes *β* oxidation in mitochondria, and upregulation of the expression of CPT1A	50 mg/kg	[55, 56]

## Data Availability

The data used in this study are available within the article.

## Abbreviations

SIRT1: Silent mating type information regulation 2 homolog-1
NAD+: Nicotinamide adenine dinucleotide
STAT3: Signal transducer and activator of transcription 3
PPAR-*γ*: Peroxisome proliferator-activated receptor gamma
FOXO: Forkhead box O
p53: Protein 53
ATP: Adenosine triphosphate
FasL: Fas ligand
Bim: Bcl-2-like protein 11
MnSOD: Manganese superoxide dismutase
Bax: Bcl-2 associated X protein
Bcl-2: B-cell lymphoma protein 2
p65: Protein 65
AST: Aspartate aminotransferase
ALT: Alanine transaminase
Nrf2: Nuclear factor-erythroid-2 related factor 2
Keap1: Kelch-like ECH-associated protein 1
H_2_O_2_: Hydrogen peroxide
MDA: malondialdehide
OH: Hydroxyl
MMP: Metaloproteinase matriks
c-Myc: Cellular-myelocytomatosis
EGFR: Epidermal growth factor receptor
MAPK: Mitogen activated protein kinase
PI3Ks: Phosphoinositide 3-kinases
Bcl-xL: B cell lymphoma-extra large
c-IAP1: Cellular inhibitor of apoptosis protein 1
Bid: BH3-domain peptide
APAF-1: Apoptotic peptidase activating factor 1
SMAC: Second mitochondria-derived activator of caspase
Cyt c: Cytochrome c
IAPs: Inhibitor of apoptosis proteins
Bak: Bcl-2 antagonist and killer
DNA: Deoxyribonucleic acid
TLR4: Toll-like receptor 4
ILs: Interleukins
TNF-*α*: Tumor necrosis factor alpha
ROS: Reactive oxygen species
RNS: Reactive nitrogen species
SOD: Super oxide dismutase
GSH: Glutathione
Toll/IL-1: Toll-interleukin-1
TRIF: TIR-domain-containing adapter-inducing interferon-*β*
MyD88: Myeloid differentiation primary response 88
AP-1: Activator protein 1
iNOS: Inducible nitric oxide synthase
COX-2: Cyclooxygenase-2
mRNA: Messenger ribonucleic acid
NO: Nitric oxide
PGE2: Prostaglandin E2
Src: Proto-oncogene tyrosine-protein kinase
Syk: Spleen tyrosine kinase
IRAK: Interleukin-1 receptor-associated kinase
TAK1: Transforming growth factor *β*-activated kinase-1
I*κ*B*α*: Inhibitor of *κ*B alpha
MKK: Mitogen-activated protein kinase kinase
JNK: c-Jun N-terminal kinase
p38: Protein 38
CD 14: Cluster of differentiation 14
HSCs: Hepatic stellate cells
*α*-SMA: Alpha-smooth muscle actin
TGF-*β*: Transforming growth factor beta
ECM: Extracellular matrix
SMAD: Suppressor of mothers against decapentaplegic
T*β*R-1 (ALK5): Transforming growth factor-beta type 1 receptor
T*β*R-II: Transforming growth factor-beta type 2 receptor
FDA: Food and drug administration
TBARS: Thiobarbituric acid reactive substances
p-ERK: Phospho-extracellular signal-regulated kinase
PIG3: p53-inducible gene 3
PTEN: Phosphatase and tensin homolog
mTOR: Mammalian target of rapamycin
NLRP3: NLR family pyrin domain containing 3
HMGB1: High mobility group box 1
CPT1A: Carnitine palmitoyltransferase 1A.
